# Supplementary material for: Inflammatory signaling differentially changes chromatin accessibility and gene expression of the PD- associated kinase LRRK2 between human and mice
Source: bioRxiv. 2026 Jan 12:2026.01.11.698894. Preprint. [Version 1] doi: 10.64898/2026.01.11.698894 (PMC12871371; doi:10.64898/2026.01.11.698894)
Supplement: Supplement 1 — Figure S1. Induction of LRRK2 by inflammatory stimuli across iPSC-derived cells. (a) LRRK2 expression and activity was assessed in iMicroglia following stimulation with LPS, IFN-ɣ, a combination of LPS and IFN-ɣ, α-synuclein preformed fibrils (PFFs), or zymosan. Among these, IFN-ɣ elicited the strongest induction of LRRK2. However, both LPS and PFFs also led to significant increases in LRRK2 expression (b) and Rab10 phosphorylation (c). *, p<0.05; ** p <0.001; ***, p<0.0001, ****, p<0.0001 by one-way ANOVA for treatment (LRRK2, F5,15 = 184.8; pRAB10, F5,15 = 334.1) with Tukey’s post-hoc test to compare all conditions. For clarity, only comparisons to control conditions (ctrl) are shown. (d-g) LRRK2 induction was evident in iMicroglia derived from other iPSC lines, KOLF2.1J (d, quantified for LRRK2 in e; ****, p<0.0001 by two-tailed t-test; t=24.9, df=4; quantified for pRAB10 in f; *, p=0.0107 by two-tailed t-test; t=4.518, df=4, n=9, three replicate cultures across three independent differentiations), WTC11 (g, quantified for LRRK2 in h; ****, p<0.0001 by two-tailed t-test; t=22.09, df=4; quantified for pRAB10 in i; ***, p =0.0003 by two-tailed t-test; t=11.68, df=4, n=9, three replicate cultures across three independent differentiations). LRRK2 induction was also observed in 35-day-old forebrain neurons (j, quantified in k; **, p<0.01 by two-tailed t-test; t=8.57, df=4, n=3 replicate cultures), but not in dopaminergic neurons (j, quantified in k; ns, p=0.5816 by two-tailed t-test; t=0.5987, df=4, n=3 replicate cultures) differentiated from the A18945 iPSC line. For all blots, each lane is a replicate culture and molecular weight markers are shown on the right. For all graphs, bars show mean values, error bars are standard deviation and individual points represent independent cultures. Figure S2. Sequence confirmation of CRISPR deletion clones. (a) Sanger sequencing of the ΔA/ΔA clone revealed a homozygous 412 bp deletion spanning chr12:40223572–40223984 (hg38). (b) [file NIHPP2026.01.11.698894v1-supplement-1.pdf]

bioRxiv preprint doi: https://doi.org/10.64898/2026.01.11.698894; this version posted January 12, 2026. The copyright holder for this preprint (which was not certified by peer review) is the author/funder. This article is a US Government work. It is not subject to copyright under 17 USC 105 and is also made available for use under a CC0 license.

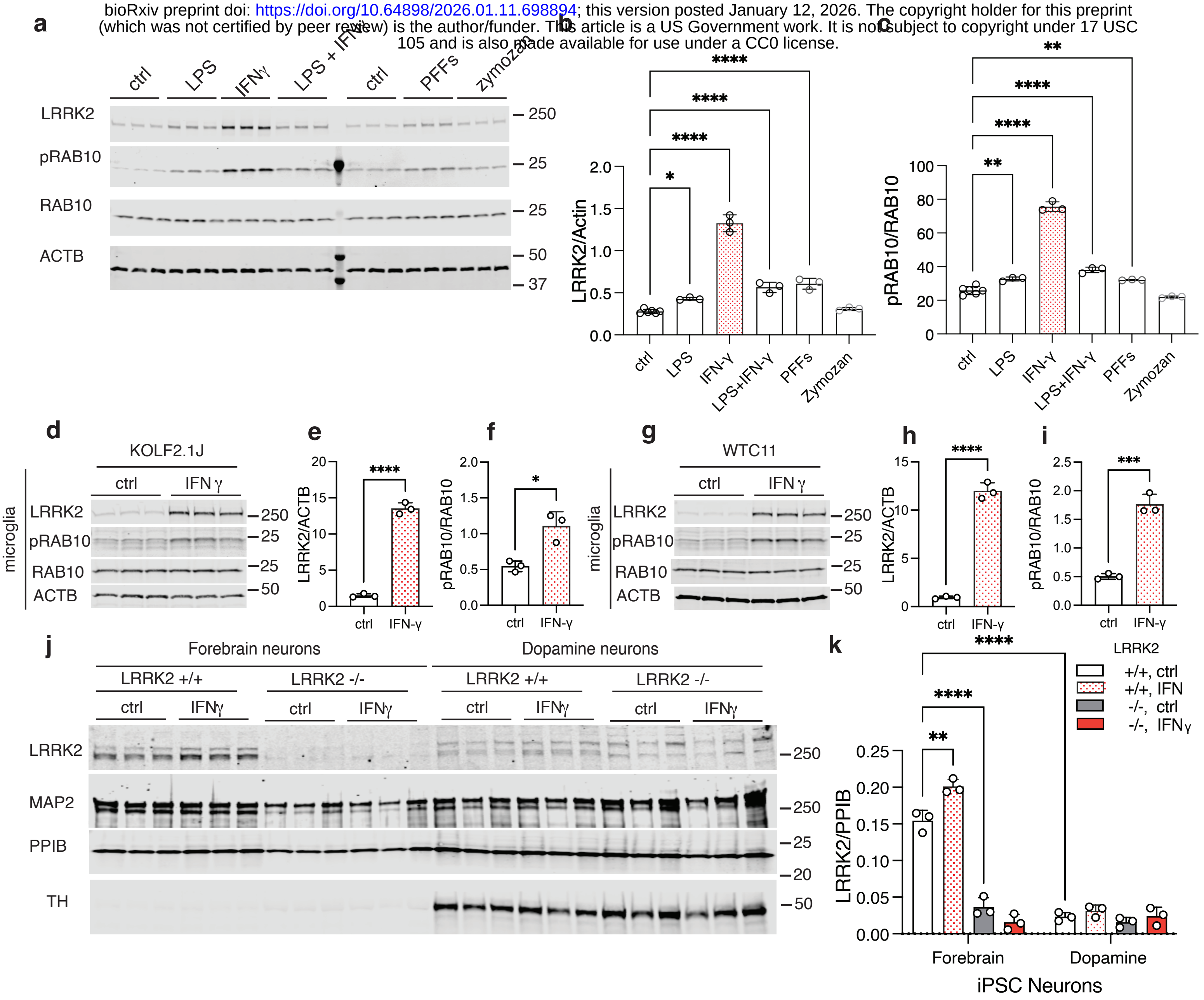

Beilina et al., Figure S1

bioRxiv preprint doi: https://doi.org/10.64898/2026.01.11.698894; this version posted January 12, 2026. The copyright holder for this preprint (which was not certified by peer review) is the author/funder. This article is a US Government work. It is not subject to copyright under 17 USC 105 and is also made available for use under a CC0 license.

a

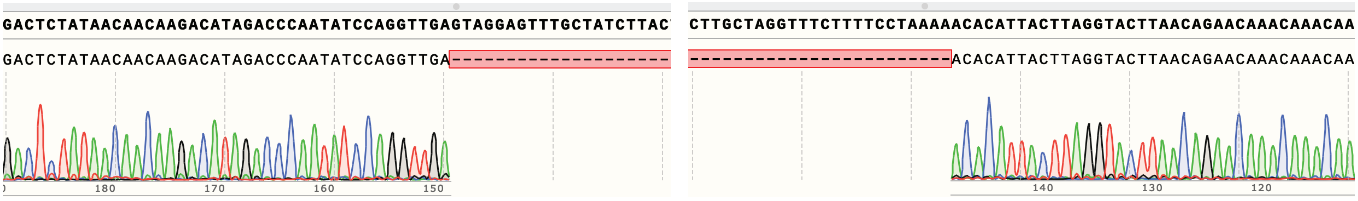

ΔA/ΔA: chr12: [40223572]–[40223984] (hg38)

b

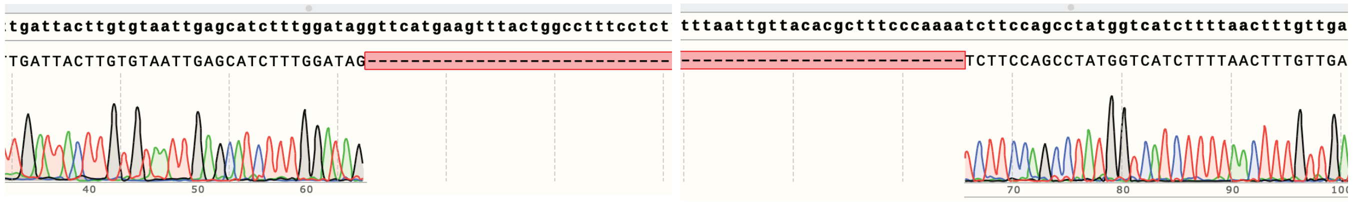

ΔB/ΔB: chr12: [40220114]–[40220646] (hg38)

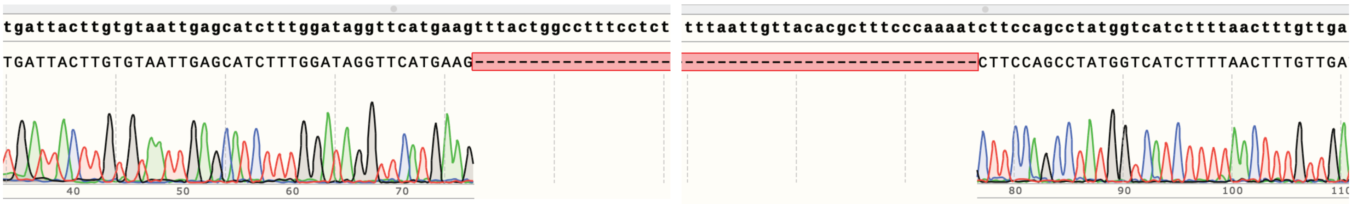

ΔB/ΔB: chr12: [40220124]–[40220647] (hg38)

Beilina et al., Figure S2

bioRxiv preprint doi: https://doi.org/10.64898/2026.01.11.698894; this version posted January 12, 2026. The copyright holder for this preprint (which was not certified by peer review) is the author/funder. This article is a US Government work. It is not subject to copyright under 17 USC 105 and is also made available for use under a CC0 license.

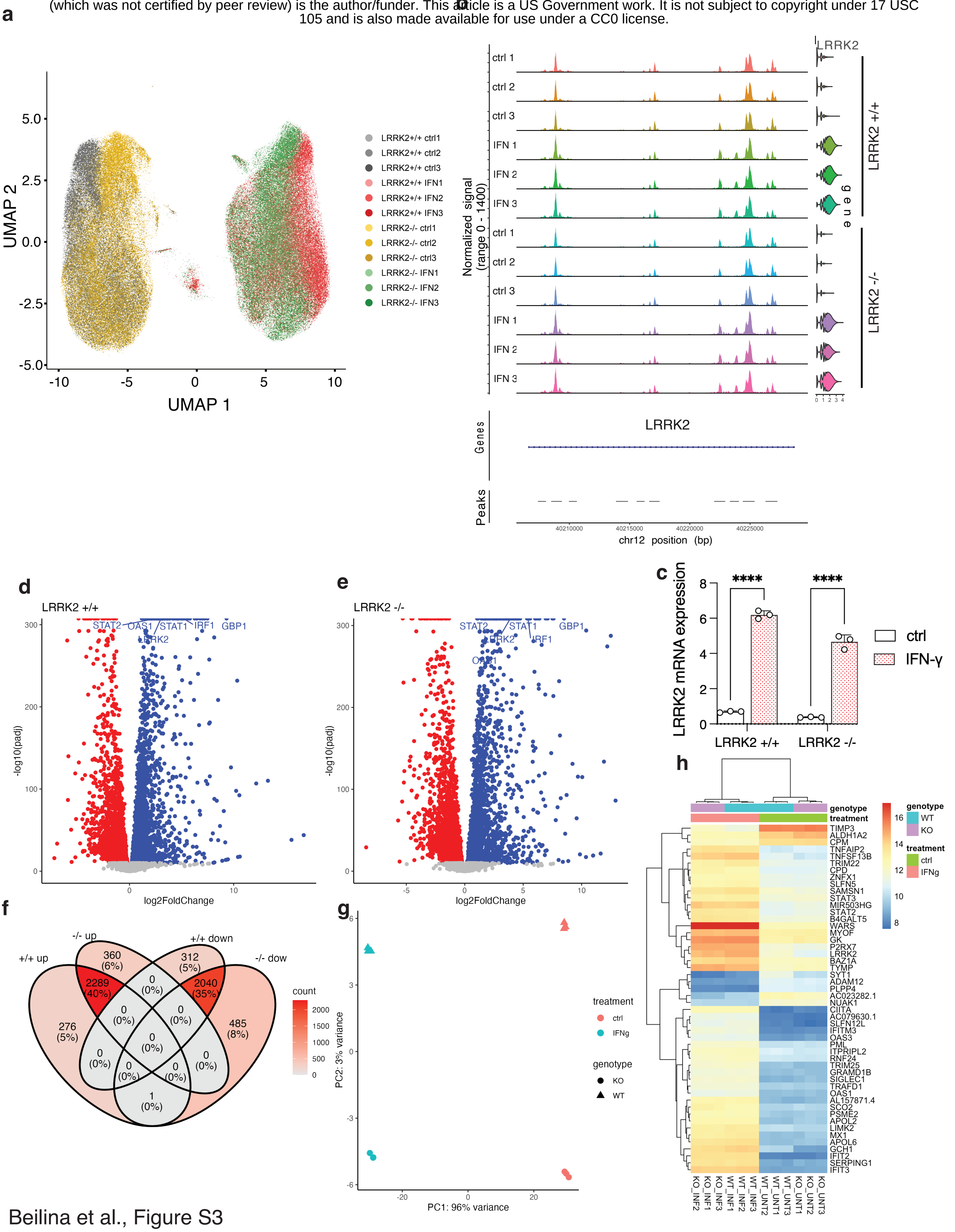

Beilina et al., Figure S3

bioRxiv preprint doi: https://doi.org/10.64898/2026.01.11.698894; this version posted January 12, 2026. The copyright holder for this preprint (which was not certified by peer review) is the author/funder. This article is a US Government work. It is not subject to copyright under 17 USC 105 and is also made available for use under a CC0 license.

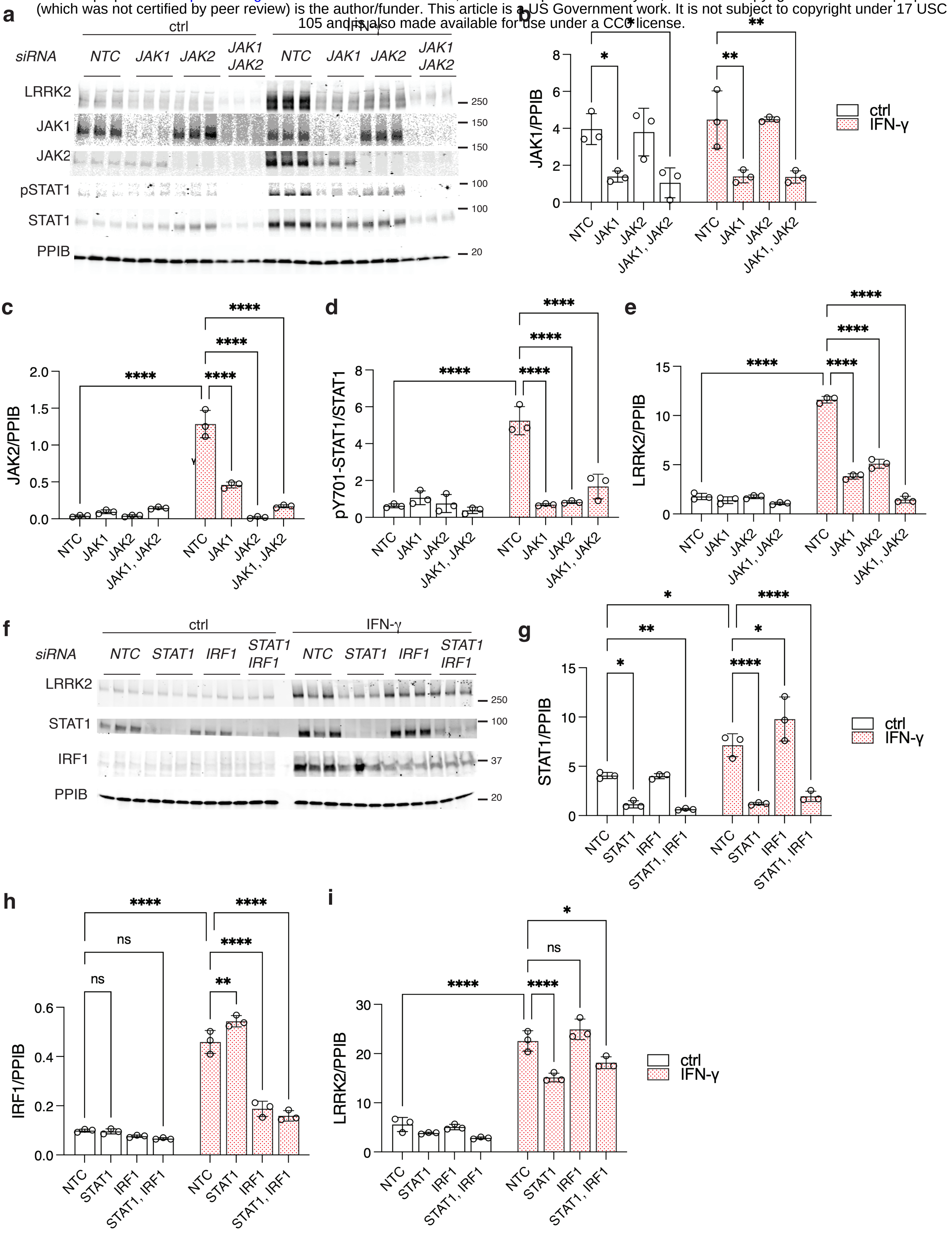

Beilina et al., Figure S4

bioRxiv preprint doi: https://doi.org/10.64898/2026.01.11.698894; this version posted January 12, 2026. The copyright holder for this preprint (which was not certified by peer review) is the author/funder. This article is a US Government work. It is not subject to copyright under 17 USC 105 and is also made available for use under a CC0 license.

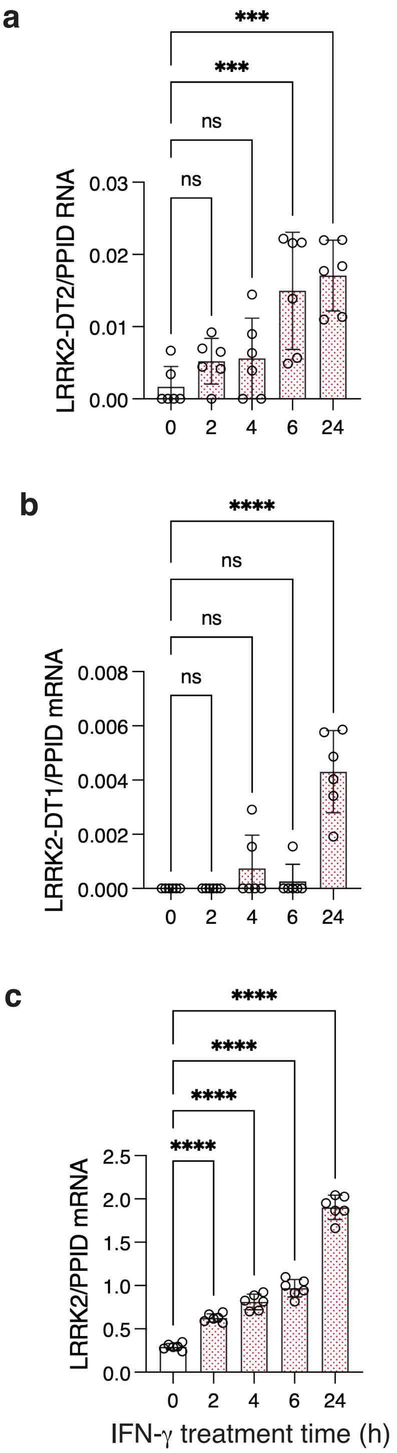

Beilina et al., Figure S5

bioRxiv preprint doi: https://doi.org/10.64898/2026.01.11.698894; this version posted January 12, 2026. The copyright holder for this preprint (which was not certified by peer review) is the author/funder. This article is a US Government work. It is not subject to copyright under 17 USC 105 and is also made available for use under a CC0 license.

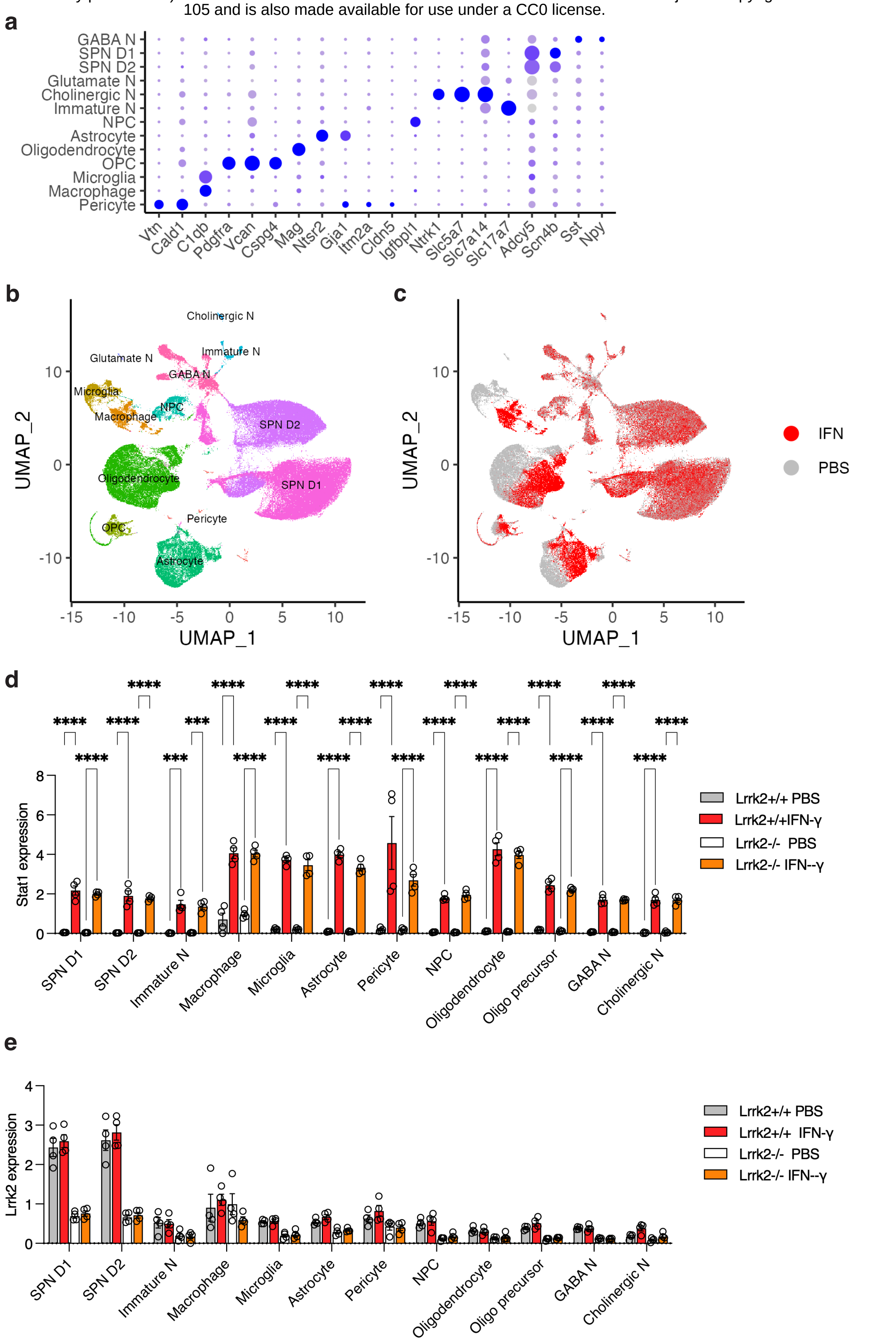

Beilina et al., Figure S6

bioRxiv preprint doi: https://doi.org/10.64898/2026.01.11.698894; this version posted January 12, 2026. The copyright holder for this preprint (which was not certified by peer review) is the author/funder. This article is a US Government work. It is not subject to copyright under 17 USC 105 and is also made available for use under a CC0 license.

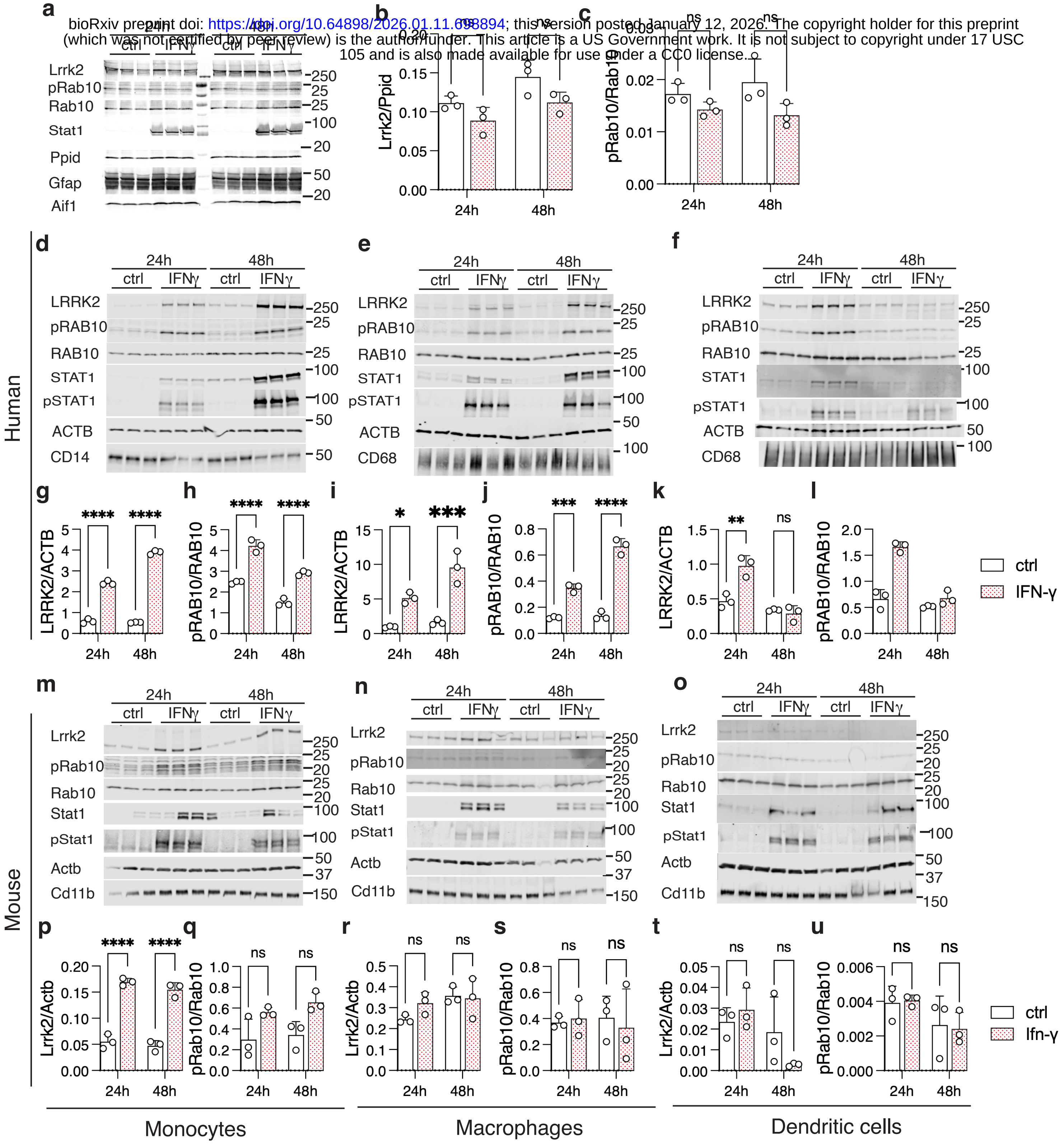

Beilina et al., Figure S7

bioRxiv preprint doi: https://doi.org/10.64898/2026.01.11.698894; this version posted January 12, 2026. The copyright holder for this preprint (which was not certified by peer review) is the author/funder. This article is a US Government work. It is not subject to copyright under 17 USC 105 and is also made available for use under a CC0 license.

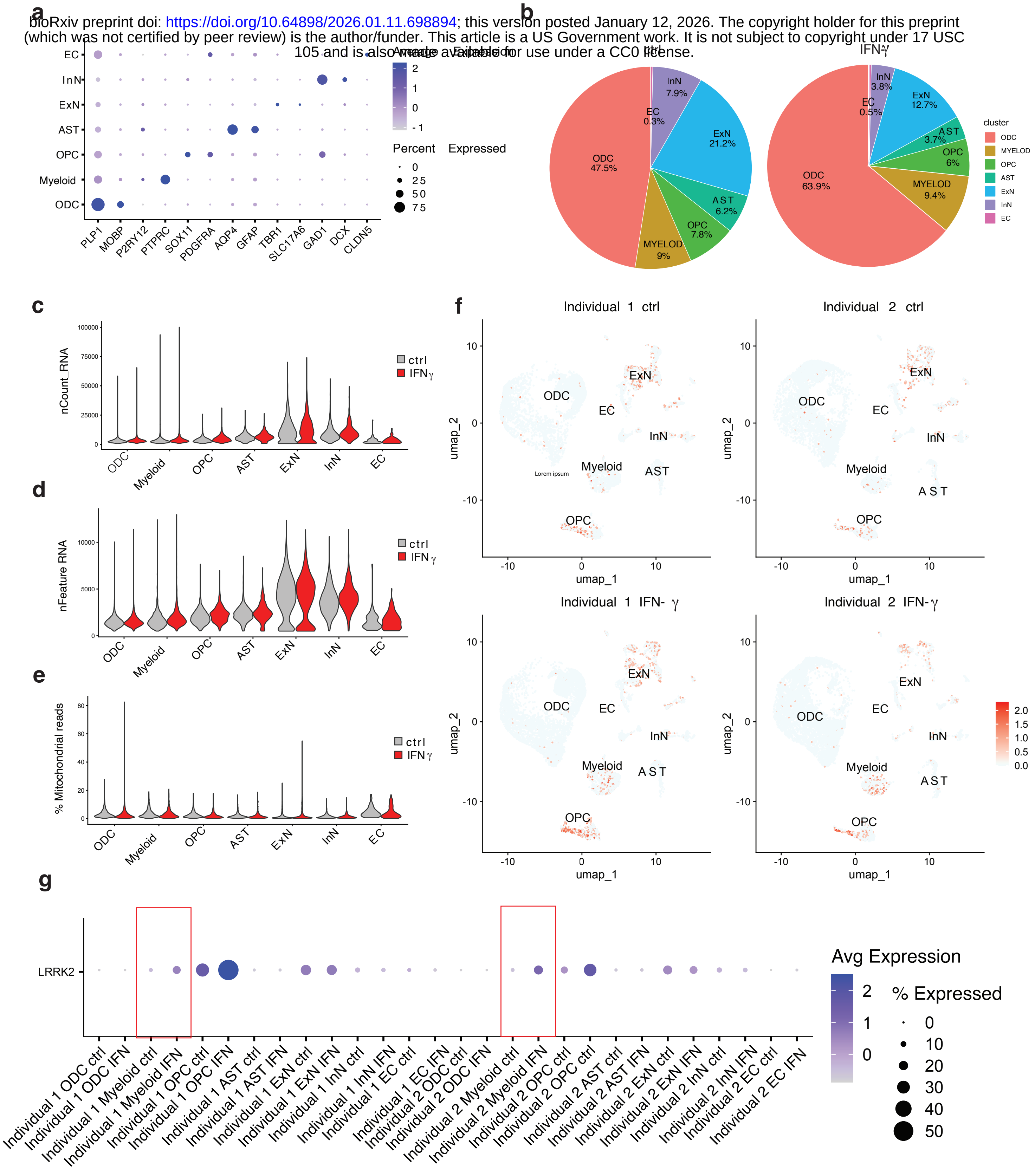

Beilina et al., Figure S8

bioRxiv preprint doi: https://doi.org/10.64898/2026.01.11.698894; this version posted January 12, 2026. The copyright holder for this preprint (which was not certified by peer review) is the author/funder. This article is a US Government work. It is not subject to copyright under 17 USC 105 and is also made available for use under a CC0 license.

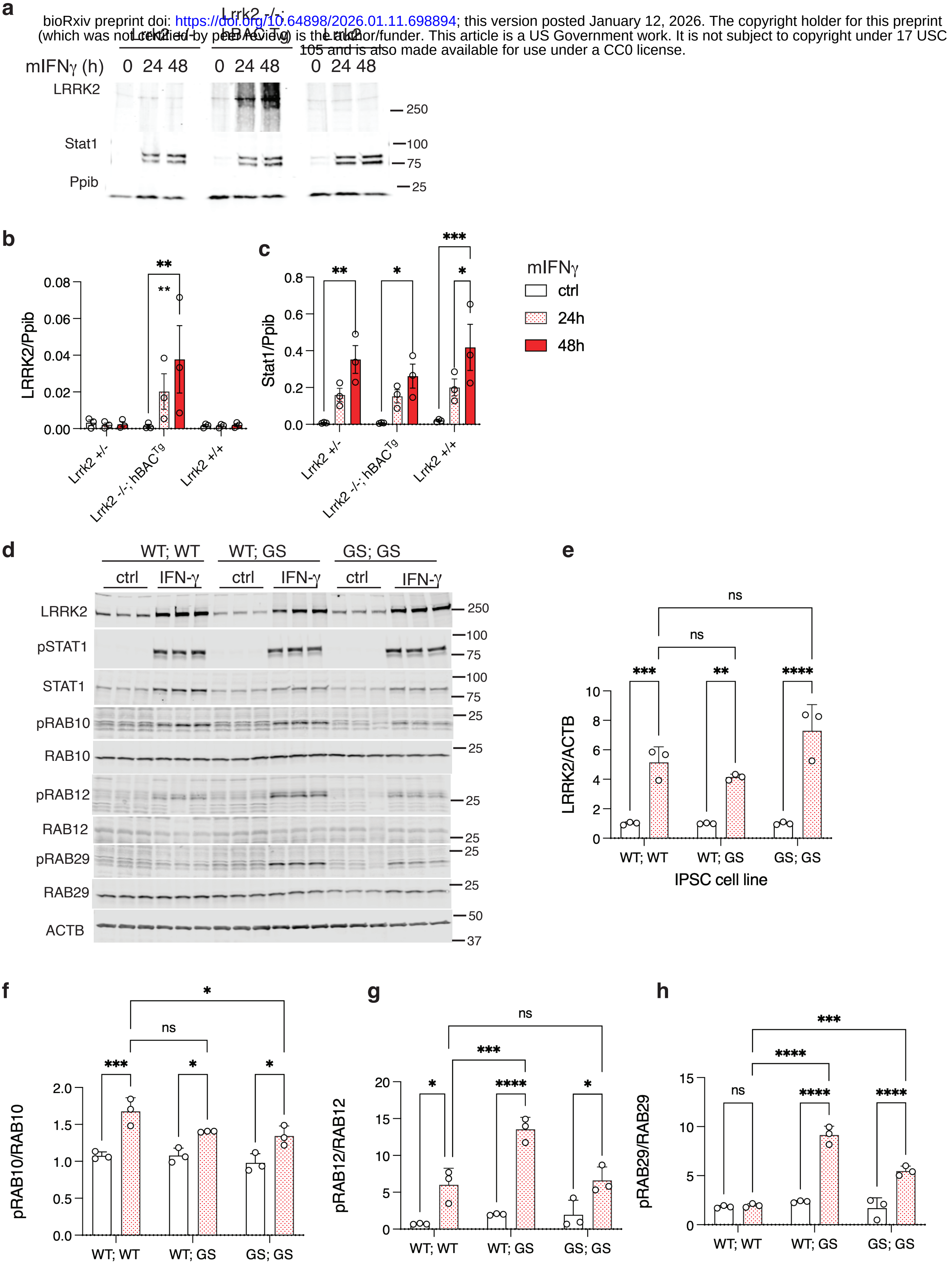

Beilina et al., Figure S9

bioRxiv preprint doi: https://doi.org/10.64898/2026.01.11.698894; this version posted January 12, 2026. The copyright holder for this preprint (which was not certified by peer review) is the author/funder. This article is a US Government work. It is not subject to copyright under 17 USC 105 and is also made available for use under a CC0 license.

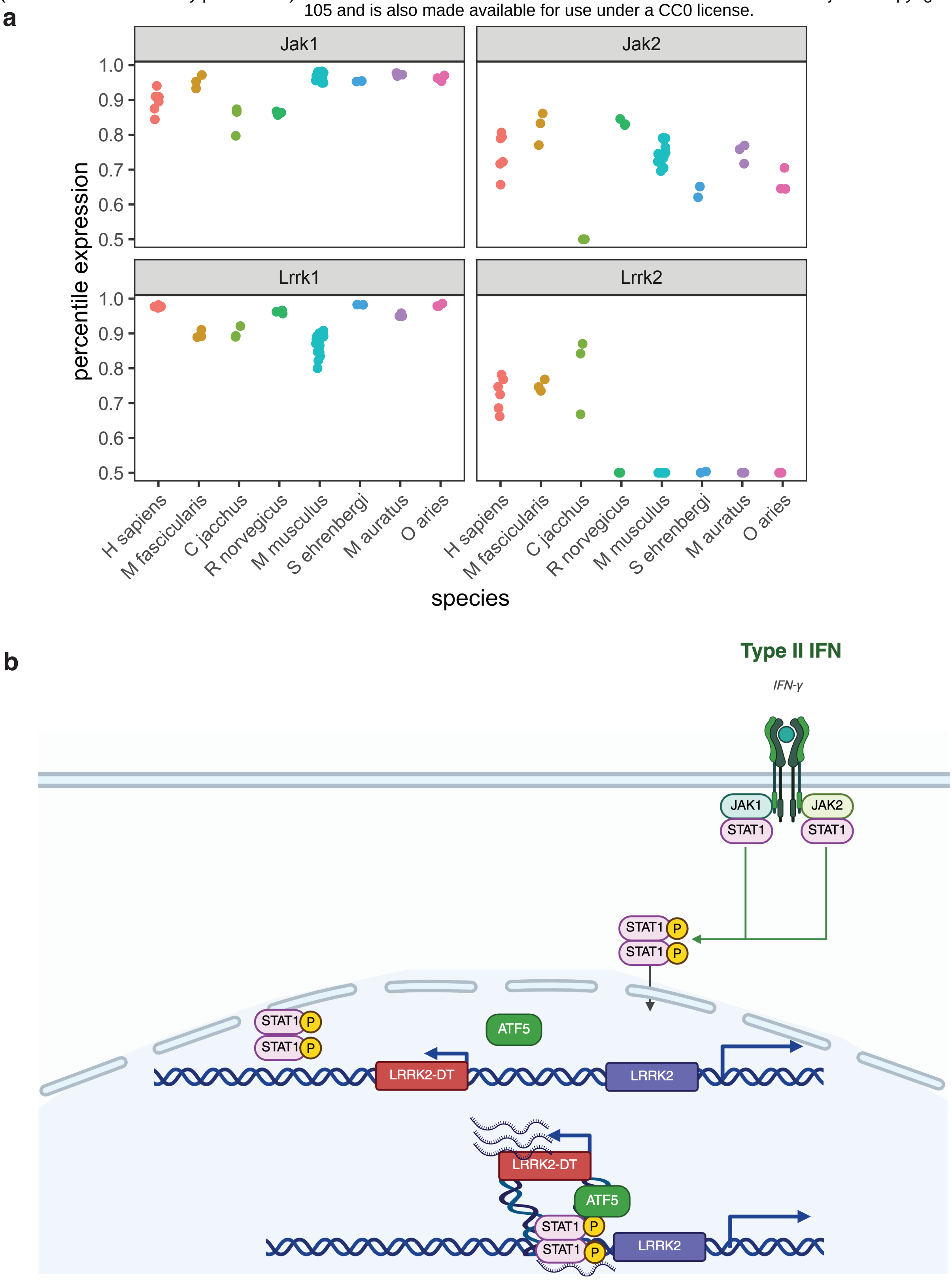

Beilina et al., Figure S10
